# Impact of Enzymatically Treated Substrate on Yellow Mealworm Development and Composition

**DOI:** 10.3390/insects16080842

**Published:** 2025-08-15

**Authors:** Michał Krzyżaniak, Olga Kosewska, Przemysław Białoskórski, Kazimierz Warmiński, Mariusz J. Stolarski, Łukasz Graban, Waldemar Lajszner, Łukasz Sikorski, Andreas Wilke, Thomas Eisele

**Affiliations:** 1 Department of Genetics, Plant Breeding and Bioresource Engineering, Faculty of Agriculture and Forestry, University of Warmia and Mazury in Olsztyn, Plac Łódzki 3, 10-724 Olsztyn, Poland; olga.kosewska@uwm.edu.pl (O.K.); przemyslaw.bialoskorski@uwm.edu.pl (P.B.); mariusz.stolarski@uwm.edu.pl (M.J.S.); lukasz.graban@uwm.edu.pl (Ł.G.); waldemar.lajszner@uwm.edu.pl (W.L.); 2 Centre for Bioeconomy and Renewable Energies, University of Warmia and Mazury in Olsztyn, Plac Łódzki 3, 10-724 Olsztyn, Poland; kazimierz.warminski@uwm.edu.pl; 3 Department of Chemistry, Faculty of Agriculture and Forestry, University of Warmia and Mazury in Olsztyn, Plac Łódzki 4, 10-721 Olsztyn, Poland; lukasz.sikorski@uwm.edu.pl; 4 Faculty of Mechanical and Process Engineering, Hochschule Offenburg, 77652 Offenburg, Germany; andreas.wilke@hs-offenburg.de (A.W.); thomas.eisele@hs-offenburg.de (T.E.)

**Keywords:** lignocellulosic feedstock, enzymatical hydrolysis, insect growth, survival, chemical composition, protein content, fatty acid content, amino acid content

## Abstract

Nowadays, insects are reared for food and feed. This idea includes the rearing of yellow mealworm (*Tenebrio molitor* L.). The study aimed to assess the effect of pretreatment of lignocellulosic materials on the growth, survival, and chemical composition of mealworm larvae. The main factor in the experiment was the type of feed. The components of the experimental mixed diets were wheat bran (control feed), enzymatically hydrolysed wheat straw pretreated with steam explosion (WES), enzymatically hydrolysed wheat straw pretreated by the organosolv method (WEO), and enzymatically hydrolysed cup plant pretreated by the organosolv method (CEO) in different combinations with wheat bran. Larval development and survival were monitored and measured. In the final bioassay, larval growth on all feeds containing 10% of pretreated lignocellulosic feed was similar to that of insects reared on the control diet. The specific growth rate of larvae reared on the WEO10 diet was significantly the highest (10.1%). The diet used to feed the insects had a significant effect on the crude protein and crude fat content in their biomass. The highest protein content was found in insects fed wheat bran and fed the CEO10 diet. Protein digestibility averaged 40.7% and did not differ statistically among diets. In conclusion, a moderate inclusion of processed lignocellulosic biomass can be used as a feed component for insect diets. Moreover, insect rearing on such substrates not only enables the utilisation of agricultural residues but also converts them into high-quality protein and fat, which can find applications in the feed, cosmetic, or food industries.

## Simple Summary

In light of ongoing climate change, it is important to undertake new initiatives that align with the concept of a circular economy. A good example of such practices is the rearing of insects to obtain valuable products, using various residues as feed. New substrates for insect rearing are constantly being sought, which helps reduce the use of materials that could otherwise be used as food or feed. Our research involved the use of processed common wheat straw (*Triticum aestivum* L.) and cup plant biomass (*Silphium perfoliatum* L.) as feed for insects. The study aimed to assess the impact of pretreating lignocellulosic materials on the growth, survival, and chemical composition of mealworm larvae (*Tenebrio molitor*). The results showed that a small addition (10–25%) of these materials did not significantly affect larval development, whereas feeding insects exclusively on these substrates had a considerable negative impact on their survival and growth. The conclusion from the study is that lignocellulosic material additives can be used in insect diets, and that their low inclusion levels could optimise production.

## 1. Introduction

Insects play a key role in ecosystems and significantly affect their biodiversity. They are one of the most widespread and diverse groups of organisms on Earth. Currently, approximately 1 million species of insects have been described and documented, which represents only a fraction of the total number of species, estimated to be between 5 and 10 million. Most of them are still waiting to be discovered and formally described [1,2]. Insects are usually associated with pests, parasites, or something unpleasant. However, they also have a positive meaning, both for us humans and for the entire biosphere [3,4,5]. Their great diversity and adaptability make them an increasingly popular subject of research in various fields, such as agriculture and biotechnology. Historically, insects have been used in traditional medicine [6,7] for the production of dyes [8] and as food in many cultures [9,10]. Today, their role is even more important in the context of global challenges related to sustainable development.

In agriculture, insects are used, among others, to control pests and reduce their numbers, and also act as bioindicators of environmental changes [11,12,13,14]. They are also important plant pollinators, and bees, especially the honeybee, play a key role in this [15]. Insects can be a source of food for humans and animals, which is particularly important in the context of global food security [16,17,18]. Insects are used as natural waste disposers, including plastics [19]. They are also an alternative resource in food production, waste management, and bio-based economies. One species that has gained particular attention is the yellow mealworm (*Tenebrio molitor* L.), an insect that can thrive on a variety of diets, providing high-value protein and lipids [20].

Studies indicate that mealworm larvae are exceptionally versatile in terms of digestion, demonstrating the ability to break down difficult-to-biodegrade materials such as plastics. This ability to break down synthetic materials shows how flexible their digestive system is, making them a particularly valuable tool in the fight against difficult-to-process waste [21,22,23]. Combined with the fact that yellow mealworm requires significantly fewer natural resources compared to traditional animal husbandry, their farming and utilisation become even more sustainable. Mealworms are not only characterised by high protein content and ease of rearing, but they are also able to consume a variety of often difficult-to-digest feed substrates, which include both agricultural by-products and more experimental, ecological substrates such as lignocellulosic biomass [24].

Crop residues contain 30–45% cellulose, 10–40% hemicellulose, and 5–25% lignin and are difficult to digest, which means that they are not a direct, high-quality raw material for animals [24]. Lignocellulose is a difficult material to process due to its complex structure, but certain insect species, and, in particular, microorganisms inhabiting their digestive tracts, including the mealworm microbiota, show some ability to degrade it, which makes them an interesting object of research [25]. However, previous studies on the use of lignocellulosic raw material for insect rearing have not yielded satisfactory results. Wang et al. [26] did not apply any pretreatment or enzymatic hydrolysis of corn stover, which resulted in poor insect development. Therefore, by using pretreated lignocellulosic biomass, it is possible to increase the efficiency of using these resources [27,28], including insect digestion, which opens up new possibilities for the valorisation of agricultural and industrial residues. The following hypotheses were adopted in the research: (1) the use of different pretreatment methods on the lignocellulosic feedstocks’ (common wheat straw (*Triticum aestivum* L.) and cup plant (*Silphium perfoliatum* L.)) biomass, as well as the enzymatic pre-hydrolysis, will facilitate the access of insects to the nutrients and will enable them to metabolise lignocellulosic biomass as feed components in insect diets; (2) the use of pretreated wheat straw and *Silphium perfoliatum* biomass in insect diets will not adversely affect the insect development and composition, especially regarding the fat and amino acids profiles.

This study aimed to investigate the development and chemical composition of *Tenebrio molitor* larvae fed with pre-processed lignocellulosic biomass and to assess the potential for valorisation of the obtained insects and residues from their rearing. By analysing how mealworms develop on such substrates, we aimed to identify new opportunities to optimise insect rearing for sustainable protein production and to strengthen the circular economy by converting residues into high-value products.

The research was divided into two main bioassays. In Experiment 1, the growth and development of insects were analysed on the main experimental diets—with or without bran—and on bran alone as a control. This experiment was conducted on a small scale. Based on the results of this bioassay, a large-scale experiment was carried out, in which insects were fed the most promising feed mixtures. In this second experiment, in addition to monitoring insect growth and development, the composition of the insects was also analysed, depending on the diet used.

## 2. Materials and Methods

### 2.1. Insect Stock

Yellow mealworm larvae used in the study came from a rearing stock maintained at the Department of Genetics, Plant Breeding and Bioresource Engineering, University of Warmia and Mazury in Olsztyn (Poland). Larvae were reared in plastic containers measuring 60 cm × 40 cm × 7 cm and were fed ad libitum with wheat bran ground to 2 mm, supplemented three times per week with fresh carrots as a water source. The colony environment was maintained at 60% relative humidity, a temperature of 26 °C and 8/16 h photoperiod. The pupae obtained from larvae rearing were sieved and placed in an oviposition box with a mesh bottom. After metamorphosis, the beetles (around 2000–2500 individuals) laid eggs to a container below. After five days, the container with eggs was removed. Newly hatched larvae were fed ad libitum with wheat bran for four weeks, after which they were sieved on 1.25 mm and 1.00 mm sieves and collected for experiments.

### 2.2. Bioassay 1: Larvae Growth and Development on Selected Feed Mixtures

The main factor in the experiment was the type of feed. The components of the experimental mixed diets were as follows:

1. Wheat bran (control feed) (WB).
2. Enzymatically hydrolysed wheat straw pretreated with steam explosion 100% (WES).
3. Enzymatically hydrolysed wheat straw pretreated by the organosolv method 100% (WEO).
4. Enzymatically hydrolysed cup plant pretreated by the organosolv method 100% (CEO).

The composition of the feed is presented in Table 1. Wheat bran was purchased from the local grain mill (Młynomag, Reszel, Poland). Wheat straw was kindly provided by Clariant GmbH (Straubing, Germany), while the cup plant was collected from the field trials conducted by UWM.

Cellulases capable of hydrolysing cellulose into β-D-glucose units were used to produce glucose from various lignocellulosic substrates. These enzymes were produced natively by Trichoderma reesei strain NRRL 15500 (ARS Culture Collection (NRRL), Peoria, IL, USA) in a fed-batch 5 L bioreactor fermentation. Following centrifugation and recovery of the first supernatant, the enzyme solution was filtered using a 0.45 µm vacuum filtration membrane. The protein concentration in the filtrate was determined using a BCA Protein Assay Kit from Thermo Fisher Scientific, Germering, Germany.

The substrates used for enzymatic hydrolysis included organosolv-pretreated wheat straw, organosolv-pretreated cup plant, and steam-exploded wheat straw provided by Clariant (Clariant Produkte GmbH, Straubing, Germany). For organosolv pretreatment of the cup plant, a 1:1 weight ratio of biomass to glycerol (86% (*w*/*v*) was used, with the addition of 2% sodium hydroxide. The mixture was autoclaved for 15 min at 121 °C and subsequently washed with water until a neutral pH was reached. All substrates were milled to a particle size of 1 mm. The organosolv-pretreated materials (wheat straw and cup plant) were processed in their moist, pressed form, and their dry matter content was determined gravimetrically. In contrast, only the steam-exploded wheat straw from Clariant was oven-dried at 105 °C prior to milling and used as a dry substrate without any further pre-treatment.

Hydrolysis was performed in a 5 L stirred bioreactor, with 510 g to 530 g of dry matter processed per batch. The thawed organosolv-pretreated substrates were pressed, and their dry mass was recorded. The enzyme concentration was dosed based on the protein content determined by the BCA assay, targeting a final concentration of 1% (*w*/*w*) relative to dry matter. The pressed moist biomass or dry substrate was added to the bioreactor along with 1 L of 50 mM citrate buffer (pH 4.5), distilled water, and the calculated volume of enzyme solution to reach a final volume of 4 L. The reactor content was pre-incubated for 2 to 3 h at 45–50 °C. During this time, the enzyme solution was also gently brought to temperature. After preincubation, the enzyme solution was added, and the hydrolysis was carried out at 50 °C for 24 h under continuous stirring. If needed, an additional 100–300 mL of distilled water was added to improve mixing.

Following hydrolysis, the reaction mixture was transferred into plastic bottles. The bioreactor was rinsed with approximately 500 mL of distilled water, which was also collected. All samples were stored at –20 °C until they were freeze-dried.

All feed materials used (including wheat bran) were additionally ground using a 2 mm sieve to break up lumps and clusters formed during processing and drying. This ensured uniform particle size across all diets. Subsequently, all feed materials were analysed for their nutritional values. However, data for mixed diets are based on calculations (Table 1). Experimental feeds were mixed in different weight proportions with wheat bran: only pre-processed lignocellulosic biomass (0% wheat bran addition), 10%, 15%, 25%, and 50% addition of experimental pre-processed lignocellulosic biomass. Wheat bran was used as a control feed as well. In this way, 16 types of diets were produced and used for insect rearing. The diets were stored at −20 °C until the start of each experimental trial.

The main experiment started with use of approx. four-week-old larvae fed with wheat bran in the stock colony. One hundred larvae were collected for every experimental box (22 cm × 13 cm × 5 cm) with aeration holes on the sides, covered by lids. Each box was provided with experimental feed and agar as a water source. Each diet was tested in three replicates in three consecutive feeding trials (n = 9). Agar was delivered twice per week in amount from 2 to 5 g twice per week (e.g., 2 g × 2 times per week). The amount depended on the larvae age.

Larvae development, survival, weight of mealworms, and the weight of remaining feed were monitored and measured every week. Each trial was run for at least eight weeks. Determination of larvae weight gain (LWG), as well individual weight (IW), was performed every seven days. For this reason, 30 randomly chosen larvae from each box were weighted and placed back to the container. Unconsumed feed with frass was also weighed (on fresh basis) and calculated by subtracting the remaining feed from feed given at the beginning of each experimental week.

Frass amount was assayed on the basis of uric acid content in unconsumed feed. Samples of unconsumed feed and frass with a minimum weight of 50 mg were taken from each box for analysis. Pure frass (without feed), collected from the stock colony, in the amount of 2–5 mg was taken as a reference. Uric acid content was determined by the electrochemical method in an alkaline extract. For this purpose, a 1–10 mg sample (frass or frass with feed) was extracted with 1 mL of sodium tetraborate solution (0.5% *w*/*w*) at 30 °C for 30 min, including 10 min in an ultrasonic bath. The filtered extract, after cooling, was analysed with a uric acid tester (electrochemical method).

At the end of each trial, the feed conversion ratio (FCR) was calculated by dividing the total mean individual consumption by the total mean individual weight gain and expressed on fresh matter basis:

FCR = weight of ingested food/weight gained.

**Figure 1:**
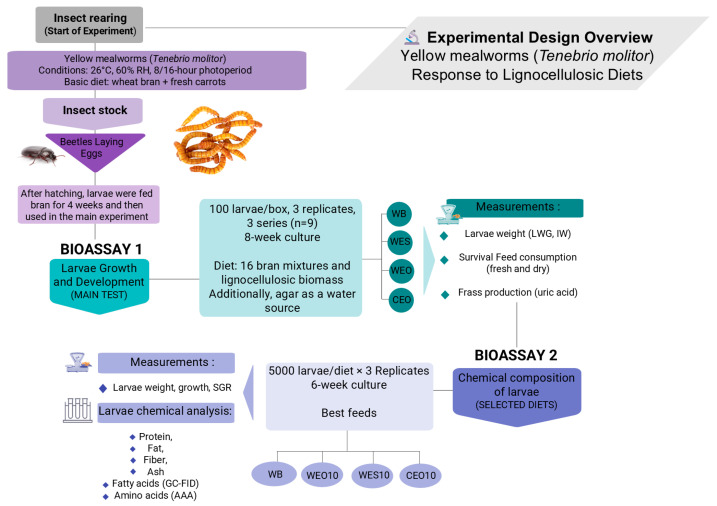
The schematic layout of the experiments in bioassay 1 and bioassay 2.

Moreover, the specific growth rate was calculated based on Crane et al. [29] using the formula:

$$100\times \left({\left(\frac{w_{2}}{w_{1}}\right)}^{\frac{1}{\Delta t}}-1\right)$$

where w_1_: initial weight of 30 larvae at time t1, w_2_: final weight of 30 larvae at time t2, and Δt: time difference between t1 (start of the experiment) and t2 (end of the experiment) expressed in days. The schematic layout of bioassay 1 is shown in Figure 1.

### 2.3. Bioassay 2: Larvae Chemical Composition and Development

This experiment aimed to rear insects on the most promising feed mixtures identified in bioassay 1. Specifically, the feeds that resulted in good larval weight gain and survival were selected: WEO10, CEO10, WES10, and WB (used as the control).

Approximately 30 day-old larvae from the stock colony were used for the experiment. For this purpose, the larvae were sieved using 1.25 mm and 1.00 mm sieves, and their mean weight was determined by weighing 30 larvae in five replicates. Based on the average weight, 5000 larvae were placed on each diet in three replicates (three boxes each diet). The larvae were kept in plastic containers (60 cm × 40 cm × 7 cm). Feed was provided at the beginning of the experiment in the amount of 1430 g per box, and agar was added at 2 × 100 g per box per week. Assuming an FCR of 2.2, it was calculated that this amount of feed should be sufficient for six weeks of rearing, after which the experiment was terminated. Subsequently, all larvae were left fasting for a minimum of 24 h and then weighted and slaughtered by freezing at −20 °C and stored for chemical analysis.

Determination of live larvae weight gain (LWG) as well individual weight (IW) were performed every seven days. For this reason, 30 randomly chosen larvae from each box were weighted (in four replicates) and placed back in the container. In the authors’ experience, in commercial yellow mealworm rearing scale, this type of measurement is justified and characterised by low labour intensity. Moreover, the coefficient of variation for such measurements was around 5%, which, according to statistical interpretation, indicates low variability within the sample. The specific growth rate was calculated as for bioassay 1. Due to the high coefficient of variation for the results of uric acid content in the frass remaining after rearing (ranging from 30 to 45%), the data concerning FCR were not presented for this experiment. The schematic layout of bioassay 2 is shown in Figure 1.

### 2.4. Chemical Analyses of Feed and Insect Depending on the Type of Diet

#### 2.4.1. Analyses of Feed Materials

The feed materials (wheat bran and the processed lignocellulosic feedstocks) were analysed in duplicate for basic nutritional values: crude protein, crude fat, crude fibre, ash, and moisture according to the methods described below. Biomass moisture was determined by the 105 °C hot-air oven method after drying to a constant weight. Crude fibre (CFib) was analysed using an ANKOM 200 Fiber Analyzer (Ankom Technology, Macedon, NY, USA). This method determines crude fibre which are the organic residue remaining after digesting with 0.255 N H_2_SO_4_ and 0.313 N NaOH (Filter Bag Technique—AOCS Standard Procedure Ba 6a-05; PN-ISO 5498:1996 [30]). Crude fat (CFat) content was analysed using the Soxhlet extraction method on the BUCHI Extraction System B-811. Approximately 2–5 g of dried and homogenised samples was placed into extraction thimbles and extracted with 150 mL of petroleum ether. In the preliminary analysis, the optimal extraction time and the degree of grinding was determined. Crude protein (CPro) content was assayed by the Kjeldahl method, with a K-435 BUCHI digestion unit and a B-324 BUCHI distiller using a protein-to-nitrogen conversion factor of 6.25 for feed and 4.76 for insects [31]. Ash was determined by incineration in an analytical muffle furnace at 550 °C for 4 h. The final component of proximate analysis is nitrogen-free extract (NFE). The nitrogen-free extract (NFE) estimates non-fibrous carbohydrates, such as soluble mono- and oligosaccharides and starches. The calculation for NFE was as follows:

NFE (% dry mass) = 100% – [CPro (% d.m) + CFat (% d.m) + CFib (% d.m) + Ash (% d.m)].

#### 2.4.2. Analyses of Insect Composition

Whole larvae from bioassay 2 were used to determine their chemical composition, depending on the diet applied. The yellow mealworm composition analysis included a proximate analysis as well as the analysis of the fatty acids and the protein amino acid content. Laboratory measurements were made for larvae obtained from separate containers. Proximate analyses were performed using the methods described in Section 2.4.1 in six replicates per diet (two replicates per box and three boxes per diet (2 × 3 = 6)).

Fatty acid (FA) composition was determined by gas chromatography with flame ionisation detection (GC-FID) of fatty acid methyl esters (FAMEs) according to the PN-EN ISO 12966-4:2015-07 standard [32]. Fatty acid derivatives were prepared using acid-catalysed transmethylation of glycerides. Fatty acid derivatives were prepared by the transesterification method using BF3 or trimethylsulfonium hydroxide (TMSH) according to the PN-EN ISO 12966-2:2017 [33] standards. The chromatographic analysis was performed using a Shimadzu GC-2014 chromatograph (Shimadzu Corporation, Kyoto, Japan) equipped with a 60 m × 0.25 mm × 0.2 μm fused silica cyanopropylpolysiloxane column with a stationary phase connected to a flame ionisation detector (FID) (PN-EN ISO 12966-4:2015 [32]). Helium (He 6.0) was used as a carrier gas. A Supelco standard solution composed of a mixture of 37 FAMEs was used for the identification of peaks. Fatty acid composition was analysed in three replicates per diet (1 × 3 = 3).

The composition of protein amino acids was determined by the method of ion exchange chromatography with post-column ninhydrin derivatization using an automatic amino acid analyser INGOS AAA 500 (Ingos s.r.o., Prague, Czech Republic) according to PN-EN ISO 13903:2006. The sample underwent chemical hydrolysis of the contained protein. Acid hydrolysates (in 6 M HCl) were used to determine most of the amino acids. Essential sulphur amino acids were determined in the acid-oxidising hydrolysates, and tryptophan in the alkaline hydrolysates, according to the procedure of the analyser manufacturer and the PN-EN ISO 13903:2006 standard [34]. Ingos amino acid standards for protein hydrolysates were used to calibrate the analyser.

Protein digestibility (PD) was determined using the standardised in vitro static digestion method by Minekus et al. [35]. Insect samples (2.5 g) underwent two-phase enzymatic hydrolysis: gastric and intestinal. The sample was first mixed with simulated salivary fluid and 0.3 M CaCl_2_. In the gastric phase, the mixture was combined with simulated gastric fluid, porcine pepsin (25,000 U·mL^−1^), 0.3 M CaCl_2_, and 1 M HCl to adjust the pH to 3.0. Final pepsin concentration was 2000 U∙mL^−1^. Incubation lasted 2 h at 37 °C. In the intestinal phase, gastric chyme was mixed with simulated intestinal fluid, pH adjusted to 7.0 with 1 M NaOH, followed by the addition of pancreatin (800 U·mL^−1^) and bile extract. Proteolytic enzyme concentration was 100 U·mL^−1^. Incubation lasted 2 h at 37 °C. The alpha amino acid content in the hydrolysate was measured by UV-Vis spectrophotometry [36,37] using o-phthaldialdehyde, dithiothreitol, and L-serine as standard. Absorbance was measured at 340 nm with a Hitachi U-1800 spectrophotometer. Protein digestibility was calculated using the following equation:

PD = CDS/CTH × 100

where CDS—concentration of free alpha-amino groups in digested samples, CTH—concentration of free alpha-amino groups in total acid hydrolysed sample.

Total acid hydrolysis was performed in 6 M HCl (110 °C, 24 h). The PD of the insects was also compared to that of casein, a highly digestible reference protein. This protein hydrolysis methodology enables the generation of dipeptides; therefore, the maximum theoretical digestibility is limited to 50% in comparison with total acid hydrolysis using HCl [36].

### 2.5. Statistical Analysis

Data from the bioassays and laboratory analyses were tested for normality (Shapiro–Wilk test) and homogeneity of variances (Levene’s test). The results showed that the data obtained from bioassay 1 did not follow a normal distribution. Therefore, differences between groups were assessed using the non-parametric Kruskal–Wallis ANOVA with a significance level of *p* < 0.05 for multiple comparisons. Group comparisons were based on global *p*-values. For the sake of clarity, only the significant differences relative to the control were presented. In the case of data collected in bioassay 2 and the chemical analyses of insects, traits such as survival, final individual weight, total biomass, SGR, protein digestibility, amino acid and fatty acid profiles followed a normal distribution. For these variables, one-way ANOVA was used with a significance level of *p* < 0.05. Homogeneous groups were identified using Tukey’s HSD test. The remaining chemical traits of the insects did not follow a normal distribution. Therefore, differences were assessed using the non-parametric Kruskal–Wallis ANOVA with a significance level of *p* < 0.05 for multiple comparisons. Group comparisons were based on global *p*-values. All statistical analyses were performed using Statistica 13 (Tibco Software Inc., Palo Alto, CA, USA).

## 3. Results

### 3.1. Bioassay 1

**Figure 2:**
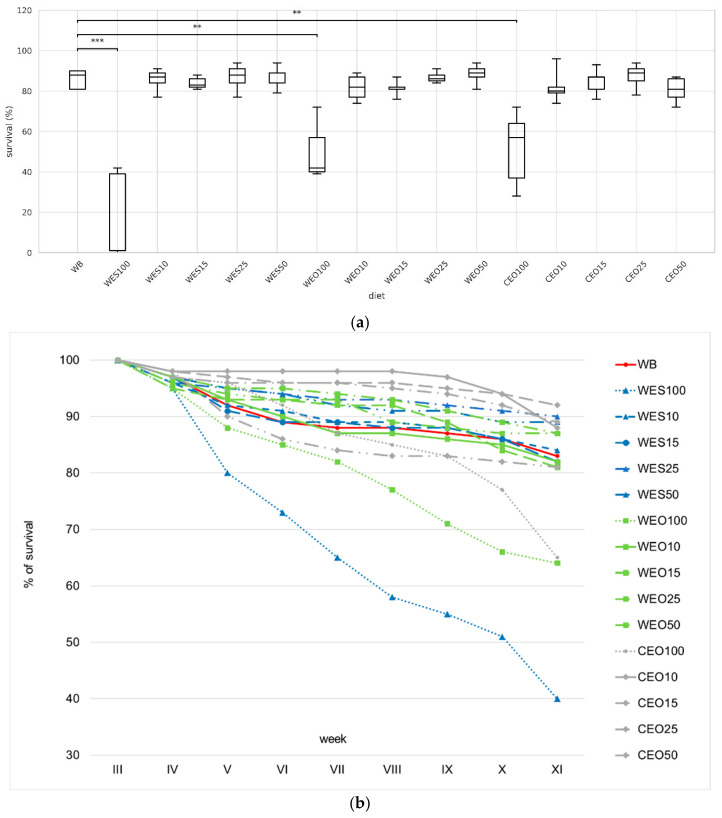
Survival of yellow mealworm larvae depending on the diet (**a**) and week (**b**). Kruskal–Wallis test: asterisks above brackets indicate significant differences between the compared groups: * *p* < 0.05, ** *p* < 0.01, *** *p* < 0.001. Diet abbreviations: WB: wheat bran; WES10-WES100: wheat bran mixed with enzymatically hydrolysed wheat straw pretreated with steam explosion; WEO10-WEO100: wheat bran mixed with enzymatically hydrolysed wheat straw pretreated using the organosolv method; CEO10-CEO100: wheat bran mixed with enzymatically hydrolysed cup plant pretreated using the organosolv method. The number following each abbreviation indicates the percentage content of the pretreated lignocellulosic material in the diet (in mixtures with wheat bran).

The use of enzymatically treated materials as feed significantly affected larval survival compared to the control (Figure 2a, Table 2). The lowest survival rate was observed for larvae reared solely on WES100 (1%), followed by WEO100 (42%) and CEO100 (57%). In contrast, diets supplemented with wheat bran, as well as the control, maintained survival at a level of 80–89%. An analysis of survival throughout the experiment shows a clear impact of WES100, WEO100, and CEO100 on larval survival as early as the fifth week of life (Figure 2b, Table S1).

**Figure 3:**
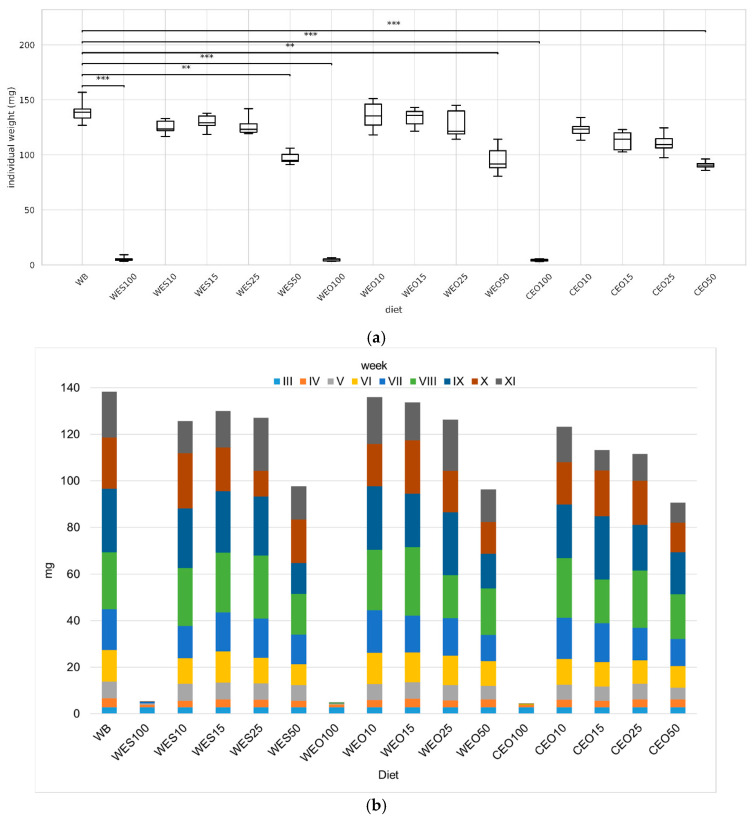
The final individual larval weight (**a**) and larva weight gain (**b**) depending on the diet. Kruskal–Wallis test: asterisks above brackets indicate significant differences between the compared groups: * *p* < 0.05, ** *p* < 0.01, *** *p* < 0.001. Diet abbreviations: WB: wheat bran; WES10-WES100: wheat bran mixed with enzymatically hydrolysed wheat straw pretreated with steam explosion; WEO10-WEO100: wheat bran mixed with enzymatically hydrolysed wheat straw pretreated using the organosolv method; CEO10-CEO100: wheat bran mixed with enzymatically hydrolysed cup plant pretreated using the organosolv method. The number following each abbreviation indicates the percentage content of the pretreated lignocellulosic material in the diet (in mixture with wheat bran).

The type of feed mixtures used significantly affected the final individual larval weight compared to the control (Figure 3a). Larvae reared on the control diet had the significantly highest weight (138.7 mg). All larvae fed diets containing 10–25% lignocellulosic material did not differ significantly in weight, which ranged from 109.3 to 136.1 mg for CEO25 and WEO15, respectively. A significant decrease in insect weight was already observed in mixtures containing 50% lignocellulosic materials (90.7–94.9 mg for CEO50 and WES50, respectively). In contrast, insects fed solely on lignocellulosic material showed almost no growth, and their weight gain over the course of the experiment was also minimal (Figure 3b, Table S1).

**Figure 4:**
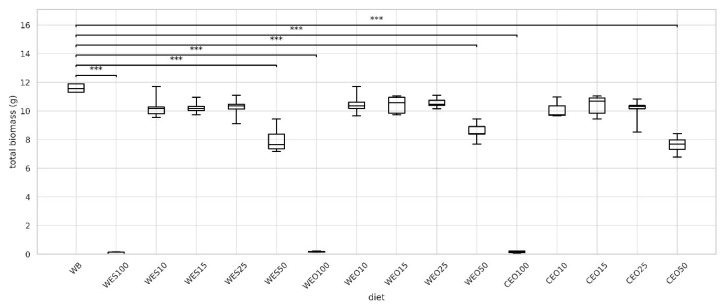
The total biomass of larvae depending on the diet. Kruskal–Wallis test: asterisks above brackets indicate significant differences between the compared groups: * *p* < 0.05, ** *p* < 0.01, *** *p* < 0.001. Diet abbreviations: WB: wheat bran; WES10-WES100: wheat bran mixed with enzymatically hydrolysed wheat straw pretreated with steam explosion; WEO10-WEO100: wheat bran mixed with enzymatically hydrolysed wheat straw pretreated using the organosolv method; CEO10-CEO100: wheat bran mixed with enzymatically hydrolysed cup plant pretreated using the organosolv method. The number following each abbreviation indicates the percentage content of the pretreated lignocellulosic material in the diet (in mixture with wheat bran).

The product of survival and final individual larval weight indicates the amount of insect biomass that can be obtained. The highest total biomass was recorded for larvae reared on the control diet (11.6 g), while the remaining insects reared on feeds containing 10–25% lignocellulosic materials had statistically similar values, ranging from 9.73 to 10.7 g (Figure 4). Mealworm larvae reared on diets with 50% lignocellulosic material showed a total biomass approximately 30% lower than the control, whereas larvae fed 100% lignocellulosic diets had 98–99% lower biomass compared to the control.

**Figure 5:**
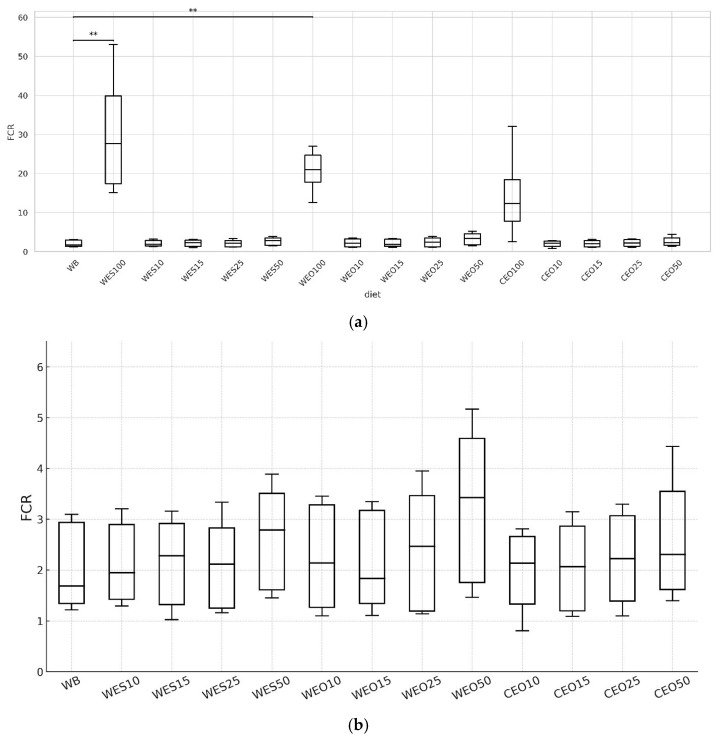
Feed conversion ratio depending on the diet used: (**a**) FCR for all diets, (**b**) for all diets except WES100, WEO100 and CEO100. Kruskal–Wallis test: asterisks above brackets indicate significant differences between the compared groups: * *p* < 0.05, ** *p* < 0.01, *** *p* < 0.001. Diet abbreviations: WB: wheat bran; WES10-WES100: wheat bran mixed with enzymatically hydrolysed wheat straw pretreated with steam explosion; WEO10-WEO100: wheat bran mixed with enzymatically hydrolysed wheat straw pretreated using the organosolv method; CEO10-CEO100: wheat bran mixed with enzymatically hydrolysed cup plant pretreated using the organosolv method. The number following each abbreviation indicates the percentage content of the pretreated lignocellulosic material in the diet (in mixture with wheat bran).

The feed conversion ratio differed significantly from the control only in the cases of the WES100 and WEO100 diets, amounting to 27.7 and 21.0, respectively (Figure 5a). The remaining diets had statistically the same FCR. The lowest FCR value was recorded for wheat bran (1.69). All other diets with a 15–50% addition of lignocellulosic material had an FCR in the range of 1.84–3.43 for WEO15 and WEO50, respectively. Despite the lack of significant differences, an upward trend in FCR was observed with increasing proportions of lignocellulosic materials in the feeds (Figure 5b). This parameter also did not differ statistically from the control in insects fed the CEO100 diet (FCR = 12.4), but this was mainly due to the high variability of results for this diet.

**Figure 6:**
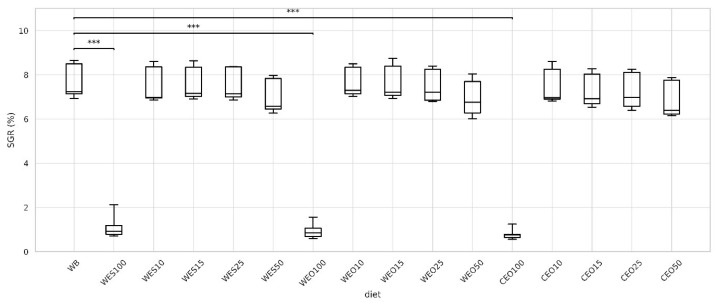
Specific growth rate of larvae depending on the diet. Kruskal–Wallis test: asterisks above brackets indicate significant differences between the compared groups: * *p* < 0.05, ** *p* < 0.01, *** *p* < 0.001. Diet abbreviations: WB: wheat bran; WES10-WES100: wheat bran mixed with enzymatically hydrolysed wheat straw pretreated with steam explosion; WEO10-WEO100: wheat bran mixed with enzymatically hydrolysed wheat straw pretreated using the organosolv method; CEO10-CEO100: wheat bran mixed with enzymatically hydrolysed cup plant pretreated using the organosolv method. The number following each abbreviation indicates the percentage content of the pretreated lignocellulosic material in the diet (in mixture with wheat bran).

The specific growth rate of yellow mealworm larvae was statistically the same for all insects fed the control diet and all diets containing up to 50% lignocellulosic materials, ranging from 6.40% to 7.30%, for larvae fed the CEO50 and WEO10 diets, respectively (Figure 6). Similarly to the FCR results, a slight downward trend in larval growth was observed with increasing proportions of lignocellulosic materials in the diet. In contrast, for diets based on sole lignocellulosic materials, the SGR during the experimental period differed significantly from the control. It was very low, ranging from 0.75% for larvae fed the CEO100 to 0.92% for WES100 diet.

### 3.2. Bioassay 2

#### 3.2.1. Growth and Development

In bioassay 2, the selected diets included wheat bran (control) and wheat bran supplemented with 10% of the enzymatically hydrolysed materials: WES10, WEO10, and CEO10. The ANOVA test (Table 3) showed that larval survival on the different diets did not differ statistically (*p* = 0.12) and averaged 99.8%, with a coefficient of variation of 4.62%.

**Figure 7:**
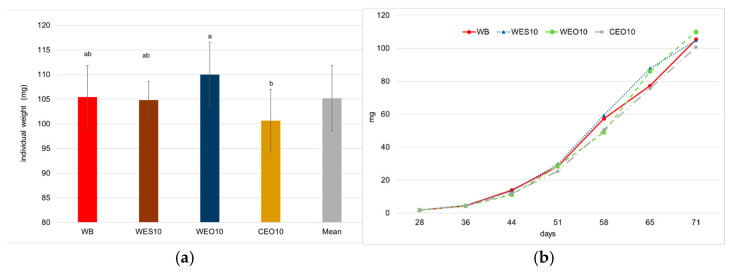
Final individual larva weight (**a**) and larva weight gain (**b**) depending on the diet. One-way ANOVA (*p* = 0.004); whiskers represent standard deviation; a, b, c, etc.: homogeneous groups according to Tukey’s HSD test. Diet abbreviations: WB: wheat bran; WES10: wheat bran and 10% of the enzymatically hydrolysed wheat straw pretreated with steam explosion 10%; WEO10: wheat bran and 10% of the enzymatically hydrolysed wheat straw pretreated by the organosolv method 100%; CEO10: wheat bran and 10% of the enzymatically hydrolysed cup plant pretreated by the organosolv method.

Individual larval weight at the end of the experiment was significantly the highest for larvae reared on the WEO10 diet (110 mg with standard deviation ±6.54 mg) (Figure 7a, Table 3). Insects reared on wheat bran and WES10 formed an intermediate group (ab), with mean weights of 105.5 ± 6.38 mg and 104.8 ± 3.86 mg, respectively. The significantly lowest individual weight was observed in larvae reared on the CEO10 diet (100.6 ± 6.35 mg). Larval growth on all feeds containing 10% lignocellulosic material during the experiment was similar to the larval weight gain of insects reared on the control diet (Figure 7b, Table S2).

**Figure 8:**
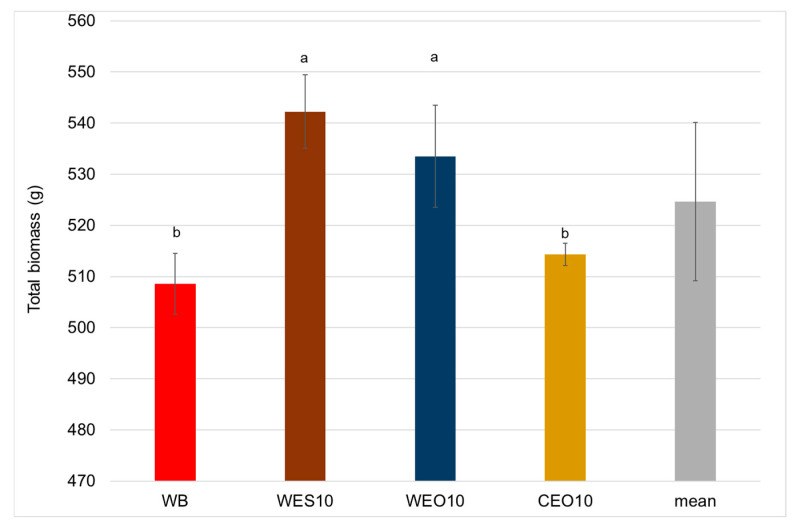
Larvae total biomass depending on the diet. One-way ANOVA (*p* = 0.004); whiskers represent standard deviation; a, b, c, etc.: homogeneous groups according to Tukey’s HSD test. Diet abbreviations: WB: wheat bran; WES10: wheat bran and 10% of the enzymatically hydrolysed wheat straw pretreated with steam explosion 10%; WEO10: wheat bran and 10% of the enzymatically hydrolysed wheat straw pretreated by the organosolv method 100%; CEO10: wheat bran and 10% of the enzymatically hydrolysed cup plant pretreated by the organosolv method.

The significantly highest total biomass was recorded for larvae reared on the WES10 and WEO10 diets, amounting to 542 and 533 g, respectively (Figure 8). The use of the remaining diets resulted in significantly lower total biomass, with 514 and 509 g for CEO10 and WB, respectively.

**Figure 9:**
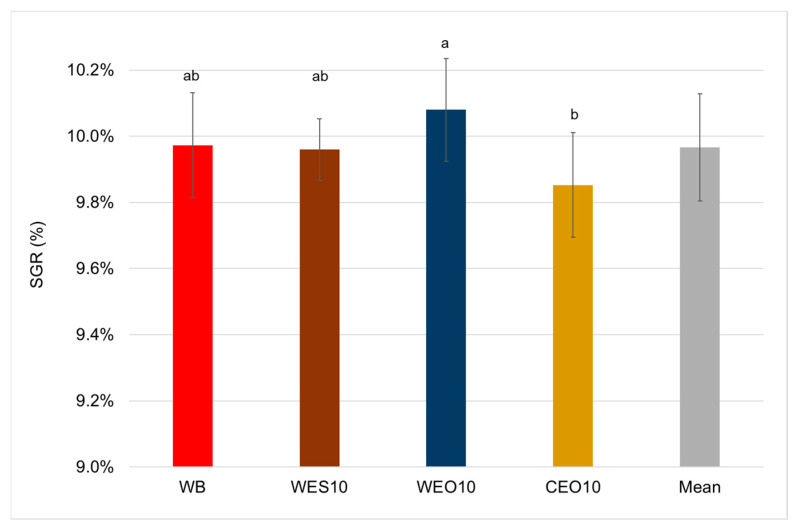
Specific growth rate of larva depending on the diet. One-way ANOVA (*p* = 0.004); whiskers represent standard deviation; a, b, c, etc.: homogeneous groups according to Tukey’s HSD test. Diet abbreviations: WB: wheat bran; WES10: wheat bran and 10% of the enzymatically hydrolysed wheat straw pretreated with steam explosion 10%; WEO10: wheat bran and 10% of the enzymatically hydrolysed wheat straw pretreated by the organosolv method 100%; CEO10: wheat bran and 10% of the enzymatically hydrolysed cup plant pretreated by the organosolv method.

The specific growth rate of larvae reared on the WEO10 diet was significantly the highest, at 10.1% (Figure 9). Larvae reared on the WB and WES10 diets formed an intermediate group in terms of this parameter, with SGR values of 9.97% and 9.96%, respectively. The significantly lowest SGR was recorded for mealworms reared on the CEO10 diet (9.85%).

#### 3.2.2. Insect Composition

**Figure 10:**
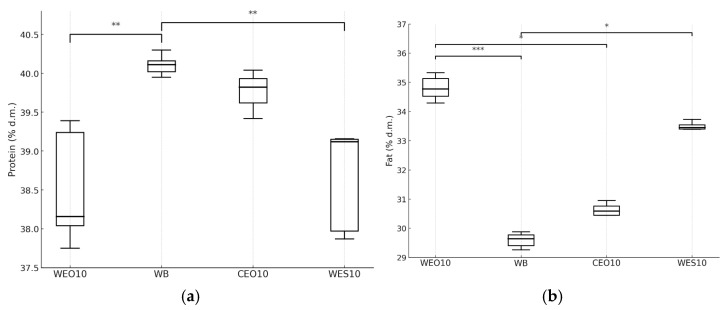
Crude protein (**a**) and crude fat content (**b**) (% d.m.) in yellow mealworm larvae depending on the diet. Kruskal–Wallis test: asterisks above brackets indicate significant differences between the compared groups: * *p* < 0.05, ** *p* < 0.01, *** *p* < 0.001. Diet abbreviations: WB: wheat bran; WES10: wheat bran and 10% of the enzymatically hydrolysed wheat straw pretreated with steam explosion 10%; WEO10: wheat bran and 10% of the enzymatically hydrolysed wheat straw pretreated by the organosolv method 100%; CEO10: wheat bran and 10% of the enzymatically hydrolysed cup plant pretreated by the organosolv method.

The diet used to feed the insects had a significant effect on the crude protein and crude fat content in their biomass. The highest protein content was found in insects fed wheat bran (40.1% d.m.) (Figure 10a, Table 4). Insects fed the CEO10 diet had a similar protein content (39.8% d.m.). Significantly lower protein levels were observed in insects fed the WES10 and WEO10 diets, at 39.1% and 38.2% d.m., respectively, compared to the control. The crude fat content in the control group was significantly the lowest, at 29.6% d.m. (Figure 10b). Insects reared on the remaining diets had from 0.95 to 5.13 percentage points more fat, for CEO10 and WEO10, respectively.

**Figure 11:**
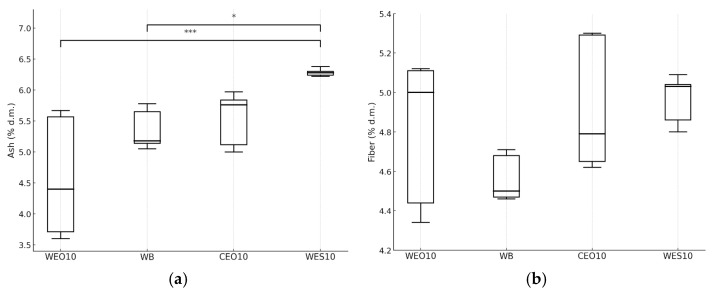
Crude ash (**a**) and crude fibre content (**b**) (% d.m.) in yellow mealworm larvae depending on the diet. Kruskal–Wallis test: asterisks above brackets indicate significant differences between the compared groups: * *p* < 0.05, ** *p* < 0.01, *** *p* < 0.001. Diet abbreviations: WB: wheat bran; WES10: wheat bran and 10% of the enzymatically hydrolysed wheat straw pretreated with steam explosion 10%; WEO10: wheat bran and 10% of the enzymatically hydrolysed wheat straw pretreated by the organosolv method 100%; CEO10: wheat bran and 10% of the enzymatically hydrolysed cup plant pretreated by the organosolv method.

The significantly highest crude ash content was found in larvae fed the WES10 diet (6.28% d.m.) (Figure 11a). A statistically similar ash content was recorded in insects fed the CEO10 diet. In contrast, the lowest ash content was found in larvae fed the WEO10 diet (4.40% d.m.). The type of diet did not affect the fibre content in the mealworm larvae biomass (*p* = 0.059) (Figure 11b). The value of this parameter ranged from 4.50% to 5.03% d.m., for insects fed WB and WES10, respectively.

**Figure 12:**
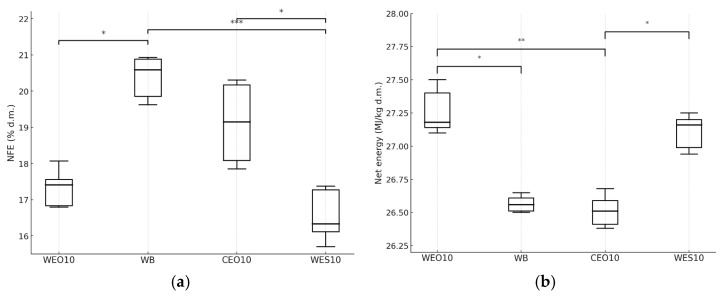
Nitrogen-free extract compounds content (**a**) (% d.m.) and net energy value (**b**) (MJ/kg d.m.) in yellow mealworm larvae depending on the diet. Kruskal–Wallis test: asterisks above brackets indicate significant differences between the compared groups: * *p* < 0.05, ** *p* < 0.01, *** *p* < 0.001. Diet abbreviations: WB: wheat bran; WES10: wheat bran and 10% of the enzymatically hydrolysed wheat straw pretreated with steam explosion 10%; WEO10: wheat bran and 10% of the enzymatically hydrolysed wheat straw pretreated by the organosolv method 100%; CEO10: wheat bran and 10% of the enzymatically hydrolysed cup plant pretreated by the organosolv method.

The nitrogen-free extract content differed significantly among insects fed the various diets. The highest content of these compounds was found in larvae fed the WB diet (20.6% d.m.) (Figure 12a), while the lowest was found in larvae fed the WES10 diet (16.3% d.m.). The net energy value was significantly the highest for insects fed the WEO10 and WES10 diets (27.2 MJ/kg d.m.) (Figure 12b). Significantly lower energy values were observed in insects fed the WB and CEO10 diets, amounting to 26.6 and 26.5 MJ/kg d.m., respectively.

**Figure 13:**
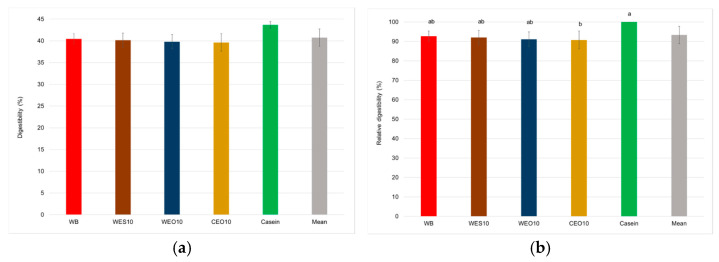
Digestibility (**a**) (%) and relative digestibility (**b**) (%), in relation to casein, of yellow mealworm protein depending on the diet. One-way ANOVA (*p* = 0.044—digestibility *p* = 0.038—relative digestibility); whiskers represent standard deviation; a, b, c, etc.: homogeneous groups according to Tukey’s HSD test. Diet abbreviations: WB: wheat bran; WES10: wheat bran and 10% of the enzymatically hydrolysed wheat straw pretreated with steam explosion 10%; WEO10: wheat bran and 10% of the enzymatically hydrolysed wheat straw pretreated by the organosolv method 100%; CEO10: wheat bran and 10% of the enzymatically hydrolysed cup plant pretreated by the organosolv method.

Protein digestibility averaged 40.7%. Statistical analysis revealed no significant differences in larval protein digestibility depending on the diet used, with values ranging from 39.6% to 40.5%, and it also did not differ significantly from the digestibility of casein (43.7%) (Figure 13a, Table 5). However, when considering relative digestibility, a significant difference compared to casein digestibility was observed for the protein of insects fed the CEO10 diet (90.8%). The relative digestibility of protein from the remaining insects formed an intermediate group ab (91.1–92.7%) (Figure 13b).

Different types of diet fed to the larvae did not significantly affect the total saturated fatty acid content (averaging 23.4% of total fatty acids). However, the type of diet did influence the content of mono- and polyunsaturated fatty acids (Table 5 and Table 6). The diets used did not affect the variation in palmitic acid (C15:0) content, but they did influence the levels of other fatty acids, such as myristic acid (C14:0) and stearic acid (C18:0). The significantly highest MUFA content was found in insects fed the WEO10 diet (47.1%), while the lowest was in those fed WB (40.9% of total fatty acids).

The diets significantly affected the content of the main fatty acid in mealworm larvae—oleic acid (C18:1). The highest level was found in larvae fed the WEO10 diet (45.3%), and the lowest in those fed WB (39.6%). The significantly highest PUFA content was found in insects fed wheat bran (36.1% of total FA), while the lowest was recorded in those fed WEO10 and WES10. The diet influenced the content of linoleic acid (C18:2) (ranging from 28.8 to 34.9% of total FA) as well as linolenic acid (C18:3). Notably, mealworm larvae fed diets with the addition of lignocellulosic materials had a significantly lower content of the latter compared to those fed the control diet (Table 5 and Table 6).

Table 7 presents the amino acid content in the protein of yellow mealworm larvae depending on the diet used. The application of different feeds significantly affected the content of threonine (highest in larvae from the CEO10 diet), cysteine (highest in WEO10), methionine (highest in WB and CEO10), tyrosine (highest in WB), phenylalanine (highest in WB), lysine (highest in WB), arginine (highest in WB), and tryptophan (highest in WB) (Table 5 and Table 7).

## 4. Discussion

In recent years, large-scale insect farming has been gaining importance as an efficient and sustainable alternative for protein production. Although it is often highlighted for its lower resource use and reduced greenhouse gas emissions compared to conventional livestock farming, in reality, insect production on such a scale involves significant operational costs—particularly related to feed and the maintenance of environmental parameters such as temperature and humidity [38,39,40].

For this reason, the main goal of the industry is to develop cheaper, efficient, and sustainable insect feeding systems. It is essential to select diets that are optimised in terms of nutrient composition, support rapid larval growth, and enhance feed conversion into insect biomass [38,41]. From this perspective, the use of agricultural residues such as straw is emerging as a promising cost-reduction strategy—the agricultural sector in the EU produces approximately 250–300 million tonnes of cereal grain annually [42]. It can be assumed that a similar amount of cereal straw is generated. These vast quantities can be converted into valuable substrates for insect farming [38,39,40].

One of the most promising examples is rice straw. Saura-Martínez et al. [38] described a complex approach involving enzymatic and physical treatment (using laccase, ultrasound, and ascorbic acid) to hydrolyse lignocellulosic fibres. As a result of this treatment, the digestibility of the straw by *T. molitor* larvae increased—leading to improved weight gain, feed conversion, and substrate utilisation efficiency. The lignocellulose hydrolysis affected approximately 13.2% of the fibres, significantly enhancing the straw’s feed value.

Other studies [40] confirm that insects can efficiently bioconvert both high- and low-quality organic substrates (including agricultural waste), which contributes to an increased protein and fat content in larval biomass without slowing growth. Moreover, key components—such as carbohydrates, fats, and fibres—have a significant impact on the properties of the final product as well as the larval development time.

In light of the findings presented above, our research also confirmed the validity of using processed lignocellulosic biomass as a feed additive for insects. Experimental feeding of yellow mealworm larvae with enzymatically processed substrates (in particular, wheat straw subjected to organosolv treatment and enzymatic hydrolysis) showed that incorporating 10–25% of this substrate into the diet allowed for survival rates and weight gains comparable to those obtained with traditional wheat bran-based diets (>85%). Moreover, parameters such as feed conversion ratio (FCR), growth rate, and the chemical composition of the insect biomass (protein and fatty acid content) remained at levels similar to the control, with no signs of dietary stress.

It is worth highlighting that larvae fed the WEO10 diet exhibited the highest specific growth rate (SGR = 7.30%) and the highest content of mono- and polyunsaturated fatty acids, making this variant particularly promising from the perspective of *T. molitor* rearing. In contrast, the use of diets based entirely on hydrolysed cellulosic material resulted in a marked decline in survival and weight gain, which is consistent with previous studies showing that diets composed exclusively of lignocellulose are the least favourable for *T. molitor* development [21].

Final larval weight and specific growth rate were the highest in the control and mixed diets (with up to 25% addition of lignocellulose-based substrates). An excessive fibre content and low levels of protein and net energy in processed lignocellulosic substrates (e.g., CEO100) limited feed utilisation. These results are consistent with the literature, which highlights the need to optimise insect feed composition to support proper development [9,43].

Recent studies indicate that the protein content in the diet of *T. molitor* should be approximately 20% [44]. A protein content close to 20%, as found in wheat bran or chicken feed, translates into high protein levels in the larvae and optimal development, as confirmed by Said and El Defrawy [45].

Our observations indicate that including lignocellulosic substrates at levels above 50% in the diet leads to significant developmental disturbances—including reduced weight gain and biomass yields lower by as much as 30–99%. In line with previous analyses [46], increasing the share of lignocellulosic substrates beyond 50% brings no benefits and often worsens the feed conversion ratio (FCR). This suggests the existence of a threshold beyond which hydrolysis and pre-treatment methods are insufficient to compensate for the low nutritional potential of substrates poor in protein and fat. A higher protein content in the diet supports growth and muscle formation, thereby leading to a lower FCR—and the opposite is also true [10].

Another factor that may negatively affect the development of insects reared on diets based on processed lignocellulosic material is the presence of toxic compounds formed during pretreatment. Both steam explosion and organosolv pretreatment can lead to the formation of toxic substances in the biomass, such as furfural, 5-HMF, and lignin-derived phenols. These compounds are known for their toxicity to fauna and may contribute to increased insect mortality and inhibited growth. A similar conclusion was reached by Theron et al. [47]. In their research, the inhibitory by-products formed during lignocellulose degradation in the pre-treatment process had a negative effect on the rearing of black soldier fly larvae, primarily due to the presence of furans.

The chemical composition and nutritional value of reared yellow mealworm larvae are significantly influenced by the composition of the feed they are grown on. The substrate affects the protein, fat, and fatty acid content of the larvae, which are crucial for their use as a protein source in animal feed and human consumption. Studies by Kröncke & Benning [48] showed that the growth performance of yellow mealworm larvae is directly dependent on the feed composition, specifically its protein content.

In our study, the crude protein content in the insects was highest when wheat bran and CEO10 were used as diets. Given that the protein content in the lignocellulosic diets was relatively similar, this generally resulted in a high—although statistically lower—protein content in insects fed the remaining diets. In other studies, yellow mealworm larvae reared on different substrates exhibited variation in protein and fat content. For instance, larvae fed on corn bran showed the highest protein content (51.33% d.m.), whereas those reared on rice bran had the lowest (46.74% d.m.) [49]. The crude protein content in mealworm larvae can reach up to 55.83%, while crude fat content may be around 25.19% [50]. However, it should be noted that a conventional protein-to-nitrogen conversion factor (Kp = 6.25) was used in that study, whereas our research adopted a factor specific to insects (4.76) [31].

Larval age is a crucial factor when assessing protein and fat content, as well as when comparing the effects of different diets on these traits. Pre-pupal larvae tend to accumulate more fat, whereas younger larvae contain less [41]. Therefore, when evaluating the impact of diet on proximate composition, this aspect should be considered. Moreover, these two traits are generally inversely correlated—higher protein levels are typically associated with lower fat content, and vice versa. Adámková et al. [51] examined the effect of rearing temperature and feed component content on larval composition and found that a higher protein diet can increase the crude protein content in insects, and the effect of feed on this parameter is much more significant than the effect of rearing temperature. To compare results between experiments, it is necessary to rear larvae in the same manner and under the same conditions, as suggested by Deruytter et al. [52].

The mineral content varies depending on the substrate. For example, larvae reared on substrates with a high ash content exhibited higher ash levels, indicating a direct correlation [53]. This finding is reflected in our results, where the highest ash content was observed in larvae reared on diets supplemented with WES and CEO—substrates with the highest crude ash content.

While feed composition significantly influences the nutritional profile of mealworm larvae, physiological processes within the larvae may alleviate the effects of certain nutrient deficiencies. For example, despite variations in feed composition, some fatty acids—such as palmitic acid and oleic acid—consistently remain prevalent, suggesting innate regulatory mechanisms in the larvae [54]. This underscores the complexity of optimising larval composition to meet specific nutritional requirements.

In our studies, protein digestibility averaged 40.7%, similar to casein (43.7%). It should be emphasised that the smallest protein hydrolysis products that can be formed are dipeptides, due to the use of endopeptidases in our analytical protocol. Therefore, the maximum theoretical protein digestibility is 50%, as only free amino groups are assayed, and if dipeptides are present, they are twice as small in the hydrolysate as free amino acids [55]. Therefore, in vitro digestibility is comparable to proteins with standard high digestibility (milk or egg proteins) [36]. In our studies, the relative digestibility of mealworm protein was over 90% compared to casein, indicating its high quality.

## 5. Conclusions

In conclusion, a moderate inclusion of processed lignocellulosic biomass can be used as a feed component in insect rearing. The use of lignocellulosic materials subjected to appropriate enzymatic pretreatment may contribute to reducing the demand for high-quality feed resources in insect farming, aligning with the zero-waste concept. Rearing *T. molitor* on such substrates not only enables the utilisation of agricultural residues but also converts them into high-quality protein and fat, which can find applications in the feed, cosmetic, or food industries.

However, challenges remain, particularly in estimating the costs of pretreatment and enzymatic hydrolysis, as well as addressing the logistical complexity at an industrial scale. Moreover, further studies are needed to assess the potential impact of toxic compounds generated during pretreatment (e.g., furfural, lignin-derived phenols) on insect survival and development. Future research should therefore continue to examine the influence of pretreatment and different cellulose hydrolysis methods on various types of agricultural residues in terms of their suitability for insect rearing. Another key aspect should be the economic evaluation of these substrates compared to conventional feed materials used in livestock production.

## Figures and Tables

**Table 1:** Proximate feed composition of analysed raw materials and feed mixtures.

Diet	Moisture (%)	Ash (% d.m.)	Protein (% d.m.)	Fat (% d.m.)	Fibre (% d.m.)	NFE (% d.m.)
WB	12.1 ± 0.02	5.56 ± 0.05	17.6 ± 0.10	3.57 ± 0.03	7.95 ± 0.49	65.3 ± 0.51
WES100	11.2 ± 0.03	6.07 ± 0.01	4.48 ± 0.02	1.02 ± 0.07	24.7 ± 0.44	63.7 ± 0.52
WEO100	10.5 ± 0.02	2.44 ± 0.01	2.18 ± 0.06	0.30 ± 0.11	41.7 ± 0.10	53.4 ± 0.14
CEO100	4.26 ± 0.02	5.66 ± 0.03	2.42 ± 0.00	1.01 ± 0.02	59.2 ± 0.41	31.7 ± 0.41
WES10	12.0	5.61	16.3	3.31	9.62	65.2
WES15	12.0	5.64	15.6	3.19	10.5	65.1
WES25	11.9	5.7	14.3	2.9	12.1	64.9
WES50	11.7	5.8	11.0	2.3	16.3	64.5
WEO10	12.0	5.25	16.07	3.24	11.3	64.1
WEO15	11.9	5.1	15.3	3.1	13.0	63.5
WEO25	11.7	4.8	13.8	2.8	16.4	62.3
WEO50	11.3	4.0	9.9	1.9	24.8	59.3
CEO10	11.3	5.57	16.09	3.31	13.1	61.9
CEO15	10.9	5.6	15.3	3.2	15.6	60.3
CEO25	10.2	5.6	13.8	2.9	20.8	56.9
CEO50	8.2	5.6	10.0	2.3	33.6	48.5

Data for the main materials are from laboratory analyses; data for mixed diets are based on calculations; ±standard deviation.

**Table 2:** Statistical results of the Kruskal–Wallis test for the analysed features of yellow mealworm larvae.

Feature	N	df	H	*p*-Value
Survival	9	15	85.50127	≤0.001
Individual weight	9	15	123.0147	≤0.001
Total biomass	9	15	117.5094	≤0.001
SGR	9	15	82.63662	≤0.001
FCR	9	15	62.60062	≤0.001

**Table 3:** Statistical results of the ANOVA test for the analysed features of yellow mealworm larvae.

Feature	N	df	F	*p*-Value
Survival	4	3	2.562	0.128
Final individual weight	4	3	5.06	0.004
Total biomass	4	3	15.78	0.001
SGR	4	3	5.0	0.004

**Table 4:** Statistical results of the Kruskal–Wallis test for the analysed chemical parameters of yellow mealworm larvae.

Feature	N	df	H	*p*-Value
Crude protein	6	3	18.99	<0.001
Crude fat	6	3	21.60	<0.001
Crude ash	6	3	16.09	0.001
Crude fibre	6	3	7.41	0.059
Nitrogen-free extract	6	3	19.42	<0.001

**Table 5:** Statistical results of the ANOVA test for the analysed chemical parameters of yellow mealworm larvae *.

Feature	N	df	F	*p*-Value
Protein digestibility	3	3	3.66	0.044
Relative digestibility	3	3	3.85	0.038
Amino acid composition:				
Thr	4	3	6.90	0.006
Cys	4	3	16.63	<0.001
Met	4	3	71.60	<0.001
Tyr	4	3	3.96	0.036
Phe	4	3	13.50	<0.001
His	4	3	3.51	0.049
Arg	4	3	4.65	0.022
Trp	4	3	10.47	0.001
Fatty acid composition:				
C12:0	4	3	14.72	0.001
C14:0	4	3	26.48	<0.001
C16:1	4	3	623.7	<0.001
C17:0	4	3	111.5	<0.001
C18:0	4	3	25.72	<0.001
C18:1	4	3	148.9	<0.001
C18:2	4	3	109.94	<0.001
C18:3	4	3	25.68	<0.001
MUFA	4	3	167.4	<0.001
PUFA	4	3	99.81	<0.001

* For amino acids and fatty acids, only significant differences (*p* ≤ 0.05) are presented.

**Table 6:** Fatty acid content of yellow mealworm larvae depending on the diet used (% of total FA).

Feed/Fatty Acid	C 12:0	C 14:0	C 15:0	C 16:0	C 16:1	C 17:0	C 17:1	C 18:0	C 18:1	C 18:2	C 18:3	C 20:0	C 20:1	SFA	MUFA	PUFA
WB	0.16 c	2.24 b	0.13 a	17.7	1.17 d	0.11 a	0.10	2.47 a	39.6 d	34.9 a	1.29 a	0.08	0.10	22.9	40.9 d	36.1 a
WEO10	0.20 a	2.99 a	0.10 b	17.8	1.61 a	0.06 b	0.08	1.94 c	45.3 a	28.8 c	0.98 b	0.07	0.08	23.2	47.1 a	29.8 c
CEO10	0.17 abc	2.54 b	0.13 a	18.1	1.39 c	0.11 a	0.05	2.22 b	42.2 c	31.8 b	1.09 b	0.06	0.08	23.4	43.7 c	32.9 b
WES10	0.18 ab	2.95 a	0.11 b	18.3	1.50 b	0.08 c	0.08	2.21 b	43.8 b	29.6 c	0.99 b	0.07	0.09	24.0	45.4 b	30.6 c
Mean	0.18	2.68	0.12	18.0	1.42	0.09	0.08	2.21	42.7	31.3	1.09	0.07	0.09	23.4	44.3	32.4

Diet abbreviations: WB: wheat bran; WES10: wheat bran and 10% of the enzymatically hydrolysed wheat straw pretreated with steam explosion 10%; WEO10: wheat bran and 10% of the enzymatically hydrolysed wheat straw pretreated by the organosolv method 100%; CEO10: wheat bran and 10% of the enzymatically hydrolysed cup plant pretreated by the organosolv method; the letters a, b, etc., show statistically homogenous groups (Tukey’s test at *p* < 0.05).

**Table 7:** Amino acid content in the protein of yellow mealworm larvae depending on the diet used (% of total protein).

Diet/Amino Acid	Asp	Thr	Ser	Glu	Pro	Gly	Ala	Cys	Val	Met	Ile	Leu	Tyr	Phe	His	Lys	Arg	Trp
WB	7.03	2.81 b	3.58	7.31	4.93	3.97	6.02	2.54 b	4.65	0.42 a	3.25	7.93	5.89 a	2.31a	3.41	4.03 a	3.99 a	0.59 a
WEO10	7.15	2.95 ab	3.74	7.60	5.14	4.11	6.06	2.89 a	4.77	0.37 b	3.24	7.94	5.59 ab	2.04 b	3.20	3.80 ab	3.69 ab	0.49 b
CEO10	7.27	3.07 a	3.87	7.46	5.21	4.19	5.99	2.54 b	4.92	0.40 a	3.21	7.74	5.48 b	2.14 b	3.21	3.72 b	3.57 b	0.46 b
WES10	7.17	2.92 ab	3.81	7.48	5.06	4.05	6.34	2.53 b	4.57	0.32 c	3.30	7.85	5.65 ab	2.06 b	3.24	3.90 ab	3.73 ab	0.41 b
Mean	7.15	2.93	3.75	7.46	5.08	4.08	6.10	2.63	4.73	0.38	3.25	7.87	5.65	2.14	3.26	3.86	3.74	0.49 b

Diet abbreviations: WB: wheat bran; WES10: wheat bran and 10% of the enzymatically hydrolysed wheat straw pretreated with steam explosion 10%; WEO10: wheat bran and 10% of the enzymatically hydrolysed wheat straw pretreated by the organosolv method 100%; CEO10: wheat bran and 10% of the enzymatically hydrolysed cup plant pretreated by the organosolv method; the letters a, b, etc., show statistically homogenous groups (Tukey’s test at *p* < 0.05)

## Data Availability

The original data presented in the study are openly available in data repository: https://bazawiedzy.uwm.edu.pl/info/researchdata/UWMd26b06e7448d42f4a62e6918a51f2c2a/ (accessed on 12 August 2025).

## Supplementary Materials

The following supporting information can be downloaded at: https://www.mdpi.com/article/10.3390/insects16080842/s1, Table S1: Survival and individual weight of yellow mealworm larvae depending on the diet and week (bioassay 1); Table S2: Individual weight of yellow mealworm larvae depending on the diet and week (bioassay 2).
